# Split Personality of a *Potyvirus*: To Specialize or Not to Specialize?

**DOI:** 10.1371/journal.pone.0105770

**Published:** 2014-08-22

**Authors:** Monica A. Kehoe, Brenda A. Coutts, Bevan J. Buirchell, Roger A. C. Jones

**Affiliations:** 1 School of Plant Biology and Institute of Agriculture, Faculty of Science, University of Western Australia, Crawley, WA, Australia; 2 Crop Protection and Lupin Breeding Branches, Department of Agriculture and Food Western Australia, Bentley Delivery Centre, Perth, WA, Australia; Virginia Tech, United States of America

## Abstract

*Bean yellow mosaic virus* (BYMV), genus *Potyvirus*, has an extensive natural host range encompassing both dicots and monocots. Its phylogenetic groups were considered to consist of an ancestral generalist group and six specialist groups derived from this generalist group during plant domestication. Recombination was suggested to be playing a role in BYMV's evolution towards host specialization. However, in subsequent phylogenetic analysis of whole genomes, group names based on the original hosts of isolates within each of them were no longer supported. Also, nine groups were found and designated I-IX. Recombination analysis was conducted on the complete coding regions of 33 BYMV genomes and two genomes of the related *Clover yellow vein virus* (CYVV). This analysis found evidence for 12 firm recombination events within BYMV phylogenetic groups I–VI, but none within groups VII–IX or CYVV. The greatest numbers of recombination events within a sequence (two or three each) occurred in four groups, three which formerly constituted the single ancestral generalist group (I, II and IV), and group VI. The individual sequences in groups III and V had one event each. These findings with whole genomes are consistent with recombination being associated with expanding host ranges, and call into question the proposed role of recombination in the evolution of BYMV, where it was previously suggested to play a role in host specialization. Instead, they (i) indicate that recombination explains the very broad natural host ranges of the three BYMV groups which infect both monocots and dicots (I, II, IV), and (ii) suggest that the three groups with narrow natural host ranges (III, V, VI) which also showed recombination now have the potential to reduce host specificity and broaden their natural host ranges.

## Introduction

*Bean yellow mosaic virus* (BYMV), genus *Potyvirus*, occurs worldwide, and has an extensive natural host range that encompasses domesticated and wild plants species, including both monocots and dicots. It causes serious diseases in a wide range of crops [1]–[4], e.g recent studies found that late infection with BYMV causes black pod syndrome (BPS) in *Lupinus angustifolius* (narrow-leafed lupin) and substantial yield losses [5], [6]. BYMV is transmitted non-persistently by many different aphid species [1], [7]. It consists of an RNA single stranded plus sense genome of about 10 kb. Its genome comprises two open reading frames (ORFs). There is one large polyprotein which is processed into ten proteins (biological characteristics linked to each in parentheses): P1 (symptomatology); HC-Pro (aphid transmission, systemic movement, suppression of gene silencing, self-interaction); P3 (plant pathogenicity); 6K1; CI (cell to cell movement); 6K2 (membrane attachment); VPg (genome replication); Nia-Pro (protein-protein interaction, cellular localization); Nib (RNA-dependent RNA polymerase, involved in replication); CP (aphid transmission, virus assembly, movement) [8], [9]. The second ORF, called PIPO, is embedded within P3, is around 180 nucleotides in length and translated in the +2 reading frame relative to the polyprotein [10]. PIPO has been linked to virulence determinacy in potyvirus resistant plants of *Pisum sativum* (pea) and long distance virus movement [11], [12].

Wylie *et al.* [13] analyzed the coat protein (CP) gene sequences of 64 BYMV isolates and based the names of the phylogenetic groups found on the types of original plant hosts that the isolates within each group came from. They proposed that these groups consisted of an ancestral generalist group with a wide natural host range and six specialist groups with narrow natural host ranges derived from the generalist group. They suggested that host specialization of BYMV had arisen within isolated crop domestication centers in different parts of the world [14], [15]. When Kehoe *et al.* [16] analyzed 40 whole BYMV genomes, they found nine phylogenetic groups which they named I–IX. The former ancestral group (called the general group) was split into three separate groups (I, II and IV). The genera the original isolation host species came from within each group were: *Lupinus, Vicia* (Fabaceae), *Freesia* (Iridaceae) and *Diurus* (Orchidaceae) in group I; *Lupinus* and *Diurus* in group II; *Gladiolus* (*Iridaceae*) in group III, *Eustoma* (Gentianacea) and *Gladiolus* in group IV, *Trifolium* (Fabaceae) and *Vicia* in group V; *Lupinus* in groups VI, VII and VIII; and *Pisum* (Fabaceae) in group IX. Thus, original host species represented in groups I, II and IV (the former general group) were from diverse origins, but those in the other groups were not. Therefore, phylogenetic group names based on natural hosts no longer seemed appropriate.

Recombination is one of the major means by which plant virus evolution and the emergence of new viruses or virus strains occurs [17]–[21]. There is evidence for high levels of recombination within the *Potyviridae* in particular [22]–[26]. Wylie and Jones [9] suggested that recombination played an important role in host specialization of BYMV following plant domestication. This suggestion was based on their analysis of seven complete genomes and 64 coat protein (CP) gene sequences. This predicted their general group to be ancestral in 12 out of 19 firm or tentative recombination patterns. However, recombination has been found to reduce host specificity and broaden natural host ranges, such as occurred with the emergence of *Maize streak virus* as an agricultural pathogen in Africa [21], [27], [28]. Therefore, given the subsequent availability of many more whole BYMV genomes and an increase in the numbers of phylogenetic groupings [16], the suggested role of recombination in the evolution of host specialization of BYMV warranted further analysis.

This research investigated the role that recombination plays in the evolution of BYMV. It examined the hypotheses that (i) recombination is associated with the expansion of natural host ranges in the three groups that contain isolates originally from both monocots and dicots (generalists), and that (ii) groups with narrow natural host ranges (specialists) might now be expanding their natural host ranges due to intermingling of strains formerly isolated from each other within crop domestication centers, resulting in recombination events creating groups with broader natural host ranges. To address these hypotheses, we undertook recombination analyses of 33 complete BYMV coding regions and two of the closely related *Clover yellow vein virus* (CYVV). These analyses included one CYVV and 13 BYMV genomes obtained as part of research on BPS [16]. As potyviruses frequently undergo recombination (see above), wherever possible, whole genome sequences should be used for recombination analysis. Therefore, our research did not include recombination analysis of BYMV and CYVV CP genes, despite many more CP sequences being available on Genbank. To determine if recombination was playing a role in their symptom expression, our research also examined the example of infection with BYMV causing BPS (late infection) or systemic necrosis (early infection) in *L. angustifolius* plants [6], [29].

## Materials and Methods

Thirty-three complete or nearly complete BYMV genomes and two CYVV genomes were retrieved from Genbank (Table 1). They were trimmed to the length of their coding regions, and aligned by Clustal W in MEGA 5.2.1 prior to analysis for recombination [30]. The RDP4 package [31] was used to detect recombination between them. Default parameters were used for the seven programs implemented within RDP: RDP [32], GENECONV [33], Bootscan [34], MaxChi [35], Chimaera [36], 3Seq [37] and SiScan [38] which included using a Bonferroni corrected *P* value cutoff of 0.05. A recombination pattern was considered to be a firm event, and genuine evidence of actual recombination, if detected by four or more of these programs, and anything less than four programs was not considered [9], [24].

**Table 1:** Bean yellow mosaic virus and Clover yellow vein virus genomes analyzed for recombination.

Accession number	Sequence ID	Phylogenetic grouping	Location^b^	Orginal isolate host	Original host type^c^	Genome Reference
HG970860	PN83A^a^	I	WA, Australia	*Lupinus angustifolius*	IC, D	[16]
HG970861	PN80A^a^	I	WA, Australia	*L. angustifolius*	IC, D	[16]
HG970852	GB17A	I	WA, Australia	*L. angustifolius*	IC, D	[16]
FJ492961	Fr	I	South Korea	*Freesia* sp.	IC, D	unpublished
HG970847	MD1	I	WA, Australia	*L. cosentinii*	NW, D	[16]
JX173278	KP2	I	WA, Australia	*Diuris magnifica*	N, M	[42]
HG970851	SP1	I	WA, Australia	*L. angustifolius*	IC, D	[16]
HG970865	AR93C^a^	I	WA, Australia	*L. angustifolius*	IC, D	[16]
HG970869	NG1	I	WA, Australia	*L. angustifolius*	IC, D	[16]
JX156423	SW3.2	II	WA, Australia	*Diuris* sp.	N, M	[42]
HG970850	MD7	II	WA, Australia	*L. cosentinii*	NW, D	[16]
HG970863	AR87C^a^	II	WA, Australia	*L. angustifolius*	IC, D	[16]
HG970855	LMBNN	II	WA, Australia	*L. angustifolius*	IC, D	[16]
HG970858	ES55C^a^	II	WA, Australia	*L. angustifolius*	IC, D	[16]
HG970854	GB32A^a^	II	WA, Australia	*L. angustifolius*	IC, D	[16]
HG970859	ES11A	II	WA, Australia	*L. angustifolius*	IC, D	[16]
AB079886	M11	III	Japan	*Gladiolus hybrida*	IC, M	[43]
AB079887	IbG	III	Japan	*Gladiolus hybrida*	IC, M	[43]
AB439729	Gla	III	Hokkaido, Japan	*Gladiolus hybrida*	IC, M	[44]
AB079888	GB2	IV	Japan	-	-	unpublished
D83749	MBGP	IV	Japan	-	-	[45]
NC003492	MB4	IV	Japan	-	-	[45]
AB439730	G1	IV	Japan	*Gladiolus hybrida*	IC, M	[44]
AM884180	Lisianthus	IV	Taiwan	*Eustoma russellianum*	IC, D	unpublished
AY192568	GDD	IV	USA	*Gladiolus sp.*	IC, M	[46]
AB439732	92-1	V	Japan	*Trifolium pratense*	IC, D	[44]
U47033	S	V	SA, Australia	*Vicia faba*	IC, D	[47]
HG970866	LP	VI	WA, Australia	*L. pilosus*	IC, D	[16]
HG970868	LPexFB	VI	WA, Australia	*V. faba*	IC, D	[16]
AB439731	90-2	VII	Japan	*V. faba*	IC, D	[44]
HG970867	FB	VII	WA, Australia	*V. faba*	IC, D	[16]
DQ641248	WLMV	VIII	Idaho, USA	*L. albus*	IC, D	[48], [49]
AB373203	CS	IX	Japan	*Pisum sativum*	IC, D	unpublished
NC003536	CYVV	n/a	Japan	*Phaseolus vulgaris*	IC, D	[50]
HG970870	CYVV AUS	n/a	NSW, Australia	*T. repens*	IC, D	[16]

a Indicates the sample originally came from a *L. angustifolius* plant with black pod syndrome.

b NSW, New South Wales; SA, South Australia; WA, Western Australia.

c Host types: D, dicot; IC, introduced cultivated plant; M, monocot; N, native plant; NW, naturalized weed.

## Results

**Figure 1:**
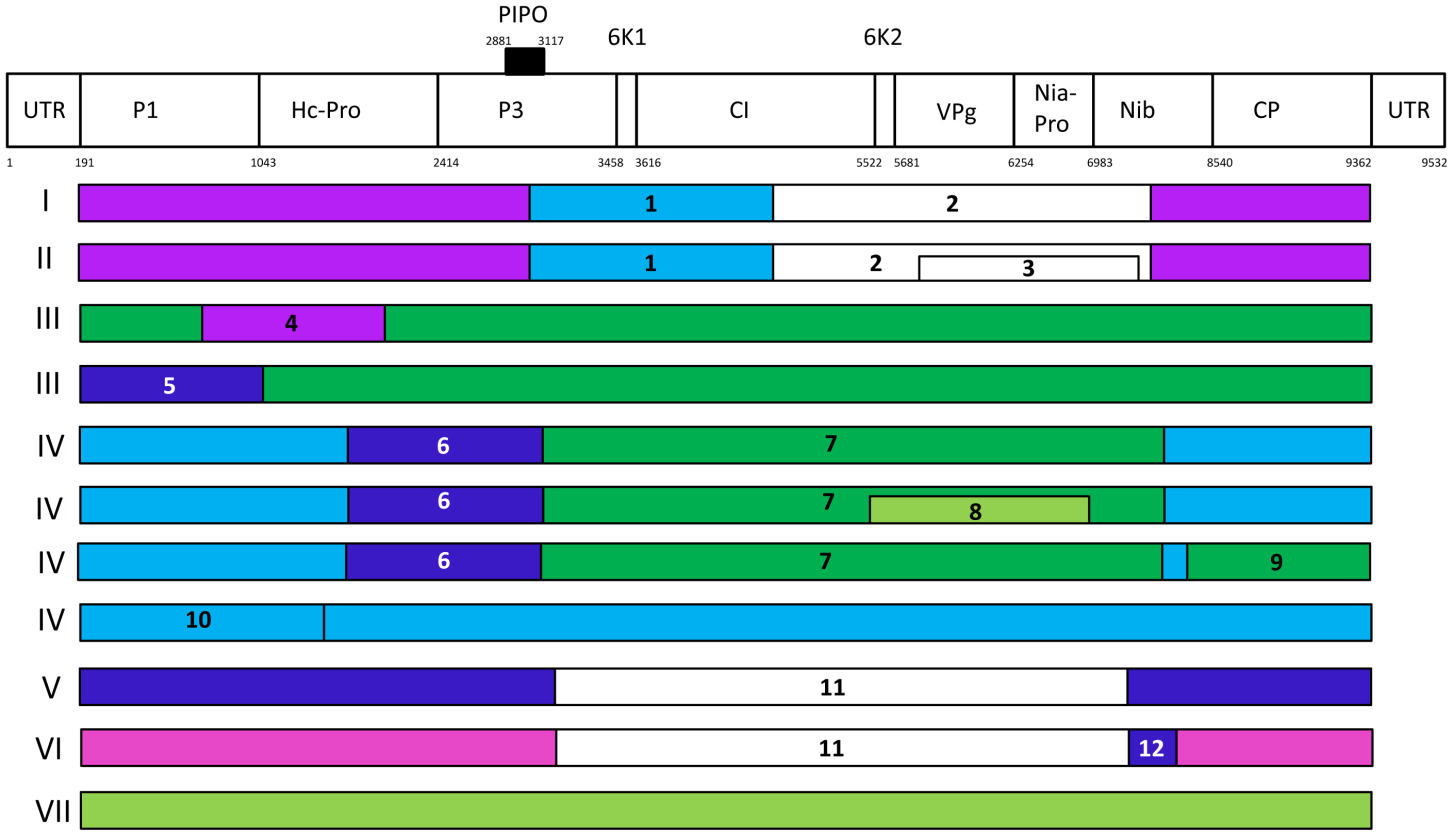
Recombination events between the coding regions of 33 *Bean yellow mosaic virus* (BYMV) and two from *Clover yellow vein virus* (ClYVV) genomes. The locations of genes in the BYMV genome are indicated by the diagram at the top of the Figure. Twelve recombination events were found, labeled 1–12. Each recombination event correlates with the event column in Table 2. Each color represents a phylogenetic group, apart from purple, which represents two such groups, I and II. The phylogenetic groupings of Kehoe *et al.* 2014a are indicated at the left hand side of the picture. The colour of each event refers to the phylogenetic grouping of its predicted parental sequences, which are detailed within Table 2. The white colour represents events whose parental sequences are unknown. The sequences analyzed were: HG970847, HG970851, HG970852, HG970860, HG970861, HG970865, HG970869, FJ492961 and JX173278 (Phylogenetic group I); HG970850, HG97054, HG970855, HG970858, HG970859, HG970863 and JX156423 (II); AB079886, AB079887 and AB439729 (III); D83749, AM884180 and AY192568 (IV); AB439732 and U47033 (V); HG970866 and HG970868 (VI); AB439731 and HG970867 (VII); DQ641248 (VIII); AB373203 (IX); NC003536 and HG970870 (ClYVV). No recombination events were detected in sequences from phylogenetic groups VII–IX or within ClYVV, but a sequence from group VII is suggested as a parental sequence for one of those from group IV.

When the complete coding regions of 33 BYMV and two CYVV isolates were analyzed, 12 firm recombination events were identified (Table 2, Fig. 1). The 16 sequences within phylogenetic groups I and II all had two recombination events across their P3, 6K1, CI, 6K2, VPg, Nia-Pro and Nib genes (events 1 and 2). The parental sequences for event 1 were from groups IV and VII. With event 2, one was unknown and the other from group IV. The seven sequences within group II also contained another, event 3 which occurred across the VPg, Nia-Pro and Nib genes. It had one unknown parental sequence and one from group I. Two of the sequences from group III (MB11 and IbG) contained event 4, which was across the P1 and Hc-Pro genes. It had parental sequences from groups II and V. The third sequence from group III (Gla) contained event 5, located in the P1 gene. Its parental sequences were from groups V and IV. Four of the sequences from group IV (MBGP, G1, Lisianthus and GB2) contained recombination events 6 and 7. Event 6 was located across the Hc-Pro and P3 genes and event 7 across the region from P3 to Nib. The parental sequences for events 6 and 7 were groups V and II, and III and VII, respectively. The sequence from GB2 contained an extra event across the region from CI to Nia-Pro. Its parental sequences were from groups VII and IV. The sequence Lisianthus had another event across the Nib and CP genes, and its parental sequences were from groups III and IV. The remaining sequence from group IV (GDD) contained event 10, located across the P1 and Hc-Pro genes with parental sequences from groups IV and I. Event 11 was found in both group V and VI sequences, and stretched from the P3 to the Nib regions. Parental sequences for event 11 were an unknown and group III. Group VI sequences (LP and LPexFB) had an additional event (event 12) in the Nib region with parental sequences from group V and an unknown sequence. There was no evidence of recombination in sequences from groups VII to IX, or in the CYVV sequences. The greatest *P*-values across all 12 recombination events ranged from 6.701×10^−7^ to 1.968×10^−160^.

**Table 2:** Recombination events in the coding regions of 33 *Bean yellow mosaic virus* and 2 *Clover yellow vein virus* genomes.

Event	Phylogenetic grouping^a^	Recombinant sequences	Programs detected by^b^	Start position in genome^c^	Genes affected	Parental sequences^d^	Parental phylogenetic group	P-value^e^
1	I, II	PN83A, PN80A, GB17A, Fr, MD1, KP2, SP1, AR93C, NG1, SW3.2, MD7, AR87C, LMBNN, ES55C, GB32A, ES11A	R, G, B, M, C, S, 3	2947–3089	P3, PIPO	GI×90-2	IV×VII	1.30×10^−78^ (3^f^)
2	I, II	PN83A, PN80A, GB17A, Fr, MD1, KP2, SP1, AR93C, NG1, SW3.2, MD7, AR87C, LMBNN, ES55C, GB32A, ES11A	R, G, B, M, C, S, 3	5203–5457	CI	unknown × MBGP	unknown × IV	3.130×10^−61^ (G)
3	II	SW3.2, MD7, AR87C, LMBNN, ES55C, GB32A, ES11A	R, G, B, M, C, S, 3	5816–5829	VPg	unknown × AR93C	unknown ×I	2.447×10^−44^ (S)
4	III	MB11, IbG	R, G, B, M, C, S, 3	1073–1099	Hc-Pro	SW3.2 ×S	II×V	3.623×10^−67^ (G)
5	III	Gla	R, G, B, M, C, S, 3	1–191 (undetermined)	5′UTR-P1	S× MBGP	V×IV	7.042×10^−110^ (G)
6	IV	MBGP, G1, Lisianthus, GB2	R, G, B, M, C, S, 3	1721–1929	Hc-Pro	S× ES11A	V×II	1.495×10^−30^ (G)
7	IV	MBGP, G1, Lisianthus, GB2	R, G, B, M, C, S	2231–2318	Hc-Pro	M11 ×90-2	III×VII	1.773×10^−55^ (S)
8	IV	GB2	R, G, B, M, C, S, 3	5506–5556	CI-6K2	90-2×G1	VII×IV	1.968×10^−160^ (G)
9	IV	Lisianthus	R, G, B, M, C, S, 3	8336	Nib	IbG×G1	III×IV	1.661×10^−79^ (S)
10	IV	GDD	R, G, B, M, C, S, 3	1–191 (undetermined)	5′UTR-P1	MBGP × PN83A	IV×I	7.249×10^−40^ (S)
11	V, VI	LP, LPexFB, S, 92-1	R, G, B, M, C, S, 3	3236–3306	P3	unknown × M11	unknown ×III	3.051×10^−47^ (G)
12	VI	Lp, LPexFB	R, B, M, C	7588–7872	Nib	92-1× unknown	V× unknown	6.701×10^−7^ (M)

a Phylogenetic grouping determined by Kehoe *et al.* (2014b).

b R, RDP; G, GENECONV; B, Bootscan; M, Maxchi; C, Chimaera; S, SiScan; 3, 3Seq.

c Numbers represent nucleotide position in the genome.

d Source of recombinant fragment. Minor parent is listed first, followed by the major parent.

e The *P*-value is the greatest value for the event in question.

f The program which detected the greatest *P*-value.

Six of the sequences analysed from groups I and II (PN83A, PN80A, AR93C, Ar87C, ES55C and GB32A) were BYMV isolates from *L. angustifolius* plants with BPS, but there was no recombination event specific to these sequences. This was also the case with three isolates (GB17A, NG1 and ES11A) from *L. angustifolius* plants with systemic necrosis within groups I and II.

## Discussion

Our research found extensive recombination amongst diverse BYMV genome sequences which is likely to have significant evolutionary implications for the virus. It revealed the presence of extensive recombination within three BYMV phylogenetic groups that include both monocots and dicots as natural hosts, supporting the suggestion that recombination leads to broadening of natural host ranges. It therefore provides evidence for the hypothesis that recombination is responsible for the wide natural host ranges of the BYMV groups that invade both dicots and monocots. It also found recombination events in three BYMV phylogenetic groups with narrow natural host ranges indicating they might now have the potential to broaden their natural host ranges. It therefore provides support for the hypothesis that groups with narrow natural host ranges might now be expanding their natural host ranges due to intermingling of strains formerly isolated from each other within crop domestication centers, resulting in recombination events and broader natural host ranges. Such a scenario would occur as a result of recombination within mixed infections between previously isolated groups. Thus, past expansion of international trade in plants and plant products would have brought BYMV isolates that evolved in isolated crop domestication centers into contact with each other resulting in recombination. These results have broader implications concerning the likely role of recombination in the evolution of plant viruses in general, especially where a distinction exists between specialist and generalist virus groups. Our research also found no indication that recombination is playing a role in producing isolates causing BPS or systemic necrosis in *L. angustifolius* plants.

Our results resemble those of Wylie and Jones [9] in that the recombination patterns found were similar. However, the dataset from our whole genome analysis was much larger (35 compared to their eight) and revealed four additional firm recombination events. Overall, we detected 12 such events across 33 BYMV and two CYVV genomes, whereas they detected eight events across seven BYMV and one CYVV genome. Their study also identified three tentative recombination events involving BYMV genomes from group IV and an unknown parent within the 3′ region of the CYVV genome. In contrast, our analysis, which excluded tentative recombination events, did not reveal any firm events involving either of the two CYVV sequences as a parent. The use of more whole genome sequences gives us greater confidence in the results.

Our results showed eight recombination events within the former general group, now groups I, II and IV (two or three events per genome), and five amongst the former specialist groups where groups III and VI had two events each and group V had one event. Groups VII–IX had no recombination events. Our findings therefore showed that the groups with the most recombination had the broadest natural host ranges that included both monocots and dicots (I, II, IV). They also found recombination within groups III, V and VI (formerly specialist groups) thereby giving them the potential to broaden their natural host ranges and thus regeneralize. However, caution is required over our interpretation as groups V–IX were only represented by one or two genomes each, so there are likely to be as yet undetected recombination events. Likewise, the limited numbers of sequences in groups V–IX also make deductions difficult regarding (i) the parents of these sequences, or (ii) the roles of these sequences as parents in other recombination events generally. Also, one of the specialist phylogenetic groups based on CP genes reported by Wylie *et al.* [13] was their canna group. Isolates from this group were unrepresented by complete genome sequences, so they could not be evaluated.

All three recombination events present in BYMV groups I and II encompass the P3, PIPO, CI and VPg regions of the genome. These regions are responsible for pathogenicity, virus long distance movement, virulence determinance towards potyvirus resistance, replication and protein-protein interactions [8], [11], [12]. However, the recombination events we detected were in isolates originally collected from symptomatic plants in field, glasshouse and experimental situations, so pathogenicity was the only characteristic that could be related to recombination. Moreover, not all viral recombinants will necessarily give rise to viable, fit variants. The nature of potyviruses is such that functions of some genes overlap with others [8]. Recombinant fitness is determined by (i) the degree to which intragenome interactions are disrupted by the event, and (ii) the divergence between the exchanged sequences, where the higher the divergence, the greater the probability that intragenome disruption will occur [39]. Recombinant virus strains or isolates with disrupted intragenome interactions are likely to be removed by negative or purifying selection, e.g. as found within the *Geminivirdae* [39]. Thus, the recombination events detected in our analysis do not reflect overall BYMV recombination rate.

Most of the complete BYMV genomes available for analysis were from Australia or Japan, so there is little scope for deductions based on geography. With the exception of one from a *Freesia* spp. in South Korea, all isolates with genomes that fit into groups I and II were collected from south-western Australia. Moreover, there are also BYMV isolates from Australia in three other groups (V, VI and VII). These findings reinforce the suggestion that BYMV arrived in Australia on at least five different occasions and that international trade, for example of bulbs and seeds, is likely responsible for the worldwide distribution of BYMV [13], [40].

It appears unlikely that any of the recombination events detected in groups I and II (events 1, 2 and 3) were responsible for the emergence of BPS as a significant disease of *L. angustifolius* caused by BYMV. No recombination event was specific to the six BYMV isolates originally from plants with BPS. Moreover, they did not differ from the four BYMV isolates originally from *L. angustifolius* plants with systemic necrosis, and one other from a plant with a susceptible reaction (non-necrotic symptoms) [6], [29], [41]. Furthermore, recombination analysis did not distinguish sequences of these *L. angustifolius* isolates from those of any other hosts in groups I or II.

As more whole genomes sequences are submitted to databases, particularly from regions of the world in which BYMV specialist groups may have originated, or where crop domestication has occurred, the picture should become clearer and we will be better able to answer the question for BYMV – to specialize or not to specialize?
